# Supplementary material for: A normal genetic variation modulates synaptic MMP‐9 protein levels and the severity of schizophrenia symptoms
Source: EMBO Mol Med. 2017 Jun 16;9(8):1100–16. doi: 10.15252/emmm.201707723 (PMC5538295; doi:10.15252/emmm.201707723)
Supplement: Supplementary file 1 — Expanded View Figures PDF [file EMMM-9-1100-s001.pdf]

Expanded View Figures

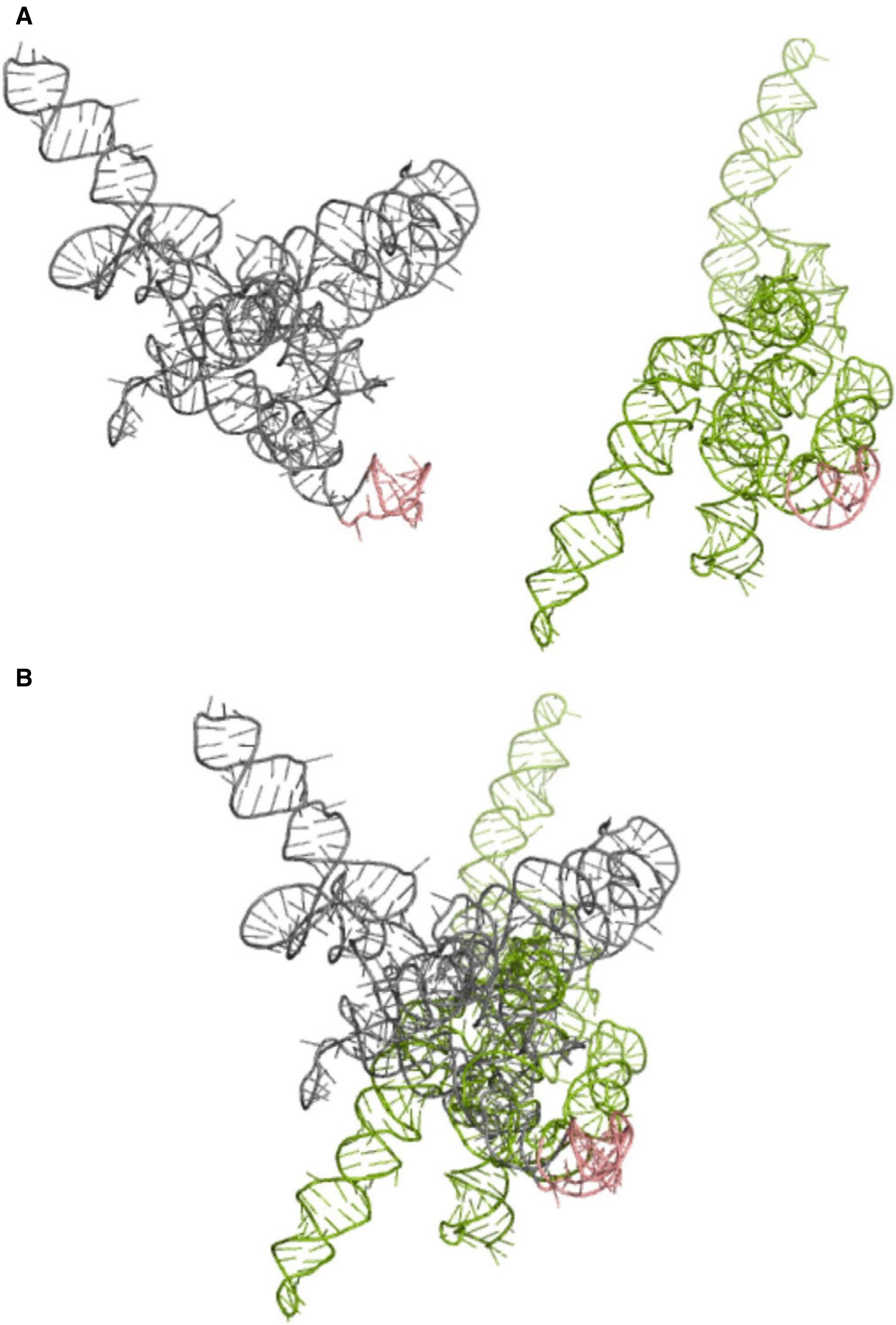

Figure EV1. 3D structure models of MMP-9_C and MMP-9_T RNA molecules.

A MMP-9_C (gray) and MMP-9_T (green) RNA structures predicted by RNAComposer based on the experimental data.

B Superposition of the MMP-9_C and MMP-9_T RNA structures from (A). The hairpin implemented in FMRP binding is shown in pink. Structures are aligned according to the hairpin implemented in FMRP binding.

 © 2017 The Authors

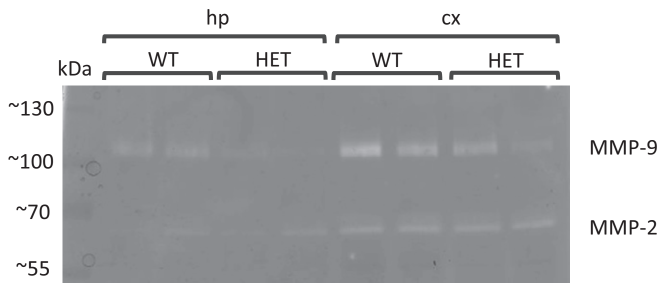

Figure EV2. The levels of MMP-9 in the brain homogenates from *Mmp-9* heterozygous (HET) and control wild-type (WT) mice.
The figure shows gel zymography from the Triton-insoluble fractions prepared from hippocampus or cortex.

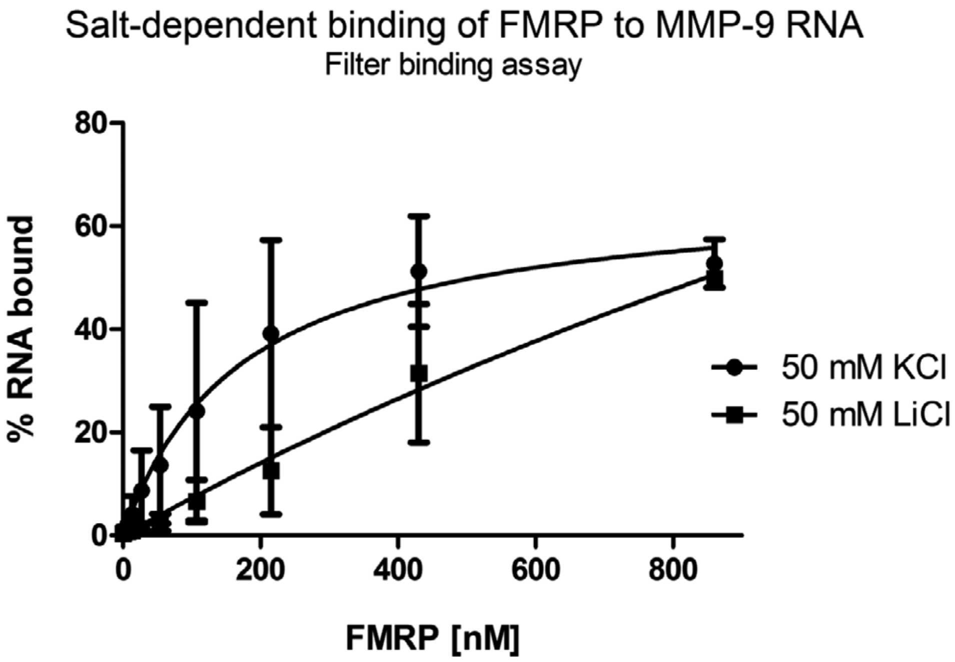

Figure EV3. Influence of lithium ions on FMRP binding to MMP-9 RNA.
Lithium ions diminish FMRP binding to the MMP-9 RNA probe, presumably by destabilizing the G-rich structure, demonstrated by the filter binding assay. The fraction of bound RNA is plotted against increasing FMRP concentrations. Each point is the mean with standard deviation of two independent experiments.

© 2017 The Authors

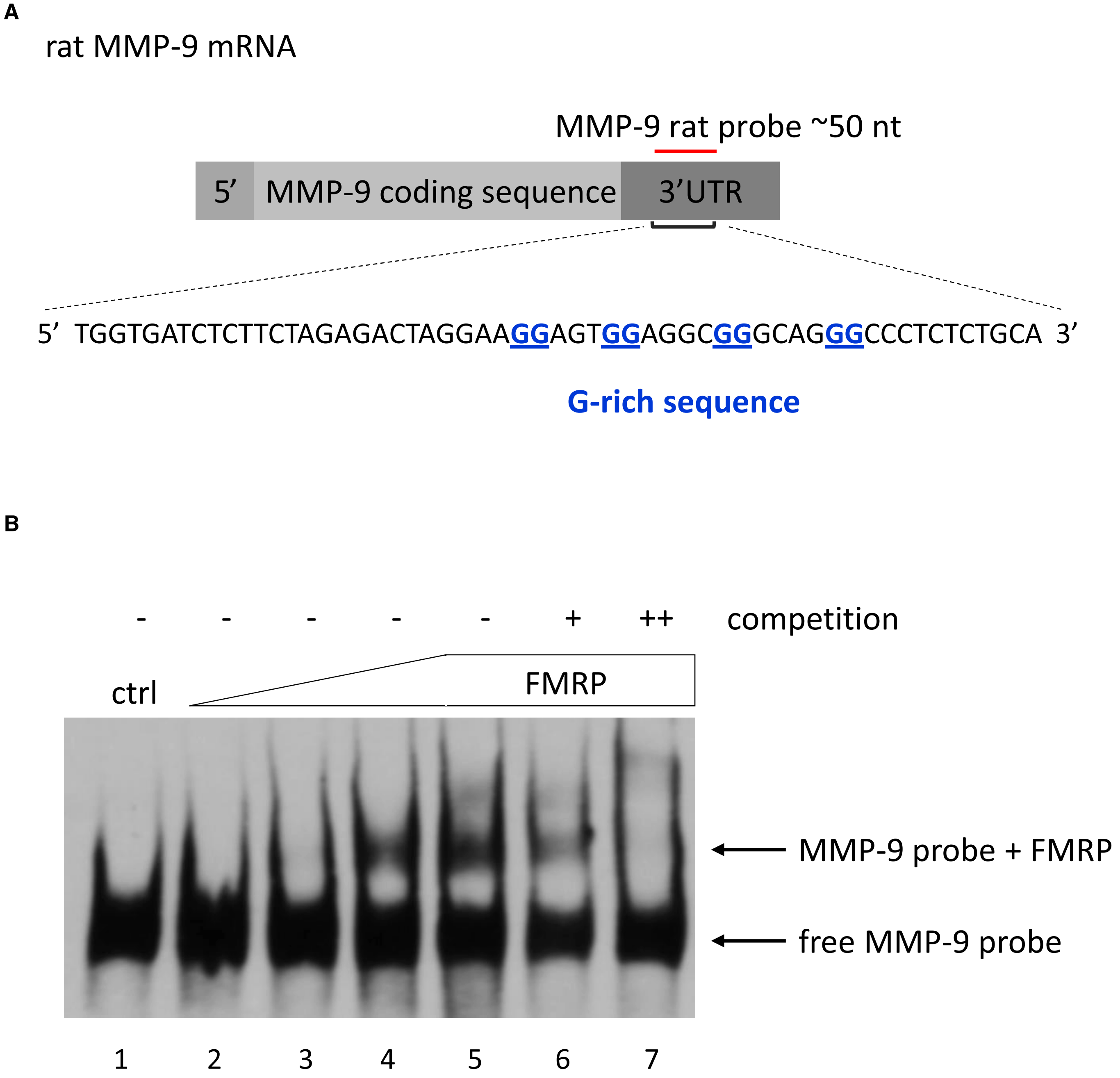

Figure EV4. FMRP binds to G-rich sequence in MMP-9 3′UTR.

A Sequence used to prepare the rat MMP-9 RNA probe that was used in the RNA electrophoretic gel-shift assay shown in (B).

B The labeled MMP-9 RNA probe was incubated in the absence (lane 1) or presence of increasing amounts of purified FMRP (70–860 nM, lanes 2–5). 20× and 200× molar excess of unlabeled probe was added as competitor to the reactions with 860 nM FMRP to confirm the specificity of the interaction (lanes 6–7, respectively). The figure shows a representative image from three independent experiments.

 © 2017 The Authors
