# Supplementary material for: A normal genetic variation modulates synaptic MMP‐9 protein levels and the severity of schizophrenia symptoms
Source: EMBO Mol Med. 2017 Jun 16;9(8):1100–16. doi: 10.15252/emmm.201707723 (PMC5538295; doi:10.15252/emmm.201707723)
Supplement: Supplementary file 2 — Table EV1 [file EMMM-9-1100-s002.docx]

|  | **Parameter analyzed** | **Exact *p-*value** | **Figure/description** |
| --- | --- | --- | --- |
| MMP-9 activity at dendritic spines | MMP-9_C vs MMP_T | *p*=0,0451 for cLTP_15min_  *p*=0,0244 for cLTP_40 min_ | Fig. 4B, page 11 |
| Percentage of mushroom spines | MMP-9_C vs MMP-9_T | *p*<0,0001 for baseline  *p*<0,0001 for cLTP_15min_  *p*<0,0001 for cLTP_40 min_ | Fig. 4D, page 12 |
|  | MMP-9_C; effect of cLTP induction | *p*=0,0179 for cLTP_15min_/baseline  *p*<0,0001 for cLTP_40min_/baseline | page 12 |
|  | MMP-9_T; effect of cLTP induction | *p*<0,0001 for cLTP_15min_/baseline *p*<0,0001 for cLTP_40min_/baseline | page 12 |
| Percentage of thin spines | MMP-9_C vs MMP-9_T | *p*=0,9962 for baseline  *p*=0,0059 for cLTP_15min_  *p*<0,0001 for cLTP_40 min_ | Fig. 4D, page 12 |
|  | MMP-9_C; effect of cLTP induction | *p*=0,9870 for cLTP_15min_/baseline  *p*=0,7208 for cLTP_40min_/baseline | page 12 |
|  | MMP-9_T; effect of cLTP induction | *p*=0,2078 for cLTP_15min_/baseline  *p*=0,0626 for cLTP_40min_/baseline | page 12 |
| Spine density | MMP-9_C vs MMP-9_T | *p*=0,8941 for baseline  *p*=0,4427 for cLTP_15min_  *p*=0,7958 for cLTP_40 min_ | page 12 |
|  | MMP-9_C effect of cLTP induction | *p*=0,3644 for cLTP_15min_/baseline  *p*=0,0211 for cLTP_40min_/baseline | page 12 |
|  | MMP-9_T effect of cLTP induction | *p*=0,0041 for cLTP_15min_/baseline  *p*=0,0056 for cLTP_40min_/baseline | page 12 |
| Head area of mushroom spines | MMP-9_C vs MMP-9_T | *p*<0,0001 for baseline  *p*<0,0001 for cLTP_15min_  *p*<0,0001 for cLTP_40 min_ | Fig. 4D, page 13 |
|  | MMP-9_C; effect of cLTP induction | *p*=0,8695 for cLTP_15min_/baseline  *p*=0,7899 for cLTP_40min_/baseline | page 13 |
|  | MMP-9_T; effect of cLTP induction | *p*=0,0003 for cLTP_15min_/baseline  *p*=0,0002 for cLTP_40min_/baseline | page 13 |
| Length of thin spines | MMP-9_C vs MMP-9_T | *p*=0,0217 for baseline  *p*=0,1836 for cLTP_15min_  *p*=0,8775 for cLTP_40 min_ | page 13 |
|  | MMP-9_C; effect of cLTP induction | *p*=0,5922 for cLTP_15min_/baseline  *p*<0,0001 for cLTP_40min_/baseline | page 13 |
|  | MMP-9_T; effect of cLTP induction | *p*=0,9887 for cLTP_15min_/baseline  *p*=0,0953 for cLTP_40min_/baseline | page 13 |
| MK-801-induced locomotor hyperactivity | Genotype effect | *p*=0.03 | Fig. 5, page 14 |
|  | Treatment genotype effect | *p*=0.000001 | Fig. 5, page 14 |
|  | Treatment effect | *p*=0.000000 | Fig. 5, page 14 |
| Filter binding | MMP-9_C vs MMP-9_T | *p*=0,3589 for 0 nM  *p*=0,0264 for 6.5 nM  *p*=0,0084 for 100 nM  *p*=0,1530 for 1000 nM | Fig. 3D, page 10 |
| REMSA | MMP-9_C vs MMP-9_T | *p*=0,3119 for 110 nM  *p*=0,0031 for 140 nM  *p*=0,2828 for 220 nM  *p*=0,0165 for 430 nM  *p*=0,1434 for 860 nM | Fig. 3C, page 10 |

**Table EV1**

Supplementary table reporting exact *p-*values from experiments described in the manuscript.
